# Regulation of TB Vaccine-Induced Airway Luminal T Cells by Respiratory Exposure to Endotoxin

**DOI:** 10.1371/journal.pone.0041666

**Published:** 2012-07-23

**Authors:** Xuerong Chen, Fangming Xiu, Carly N. Horvath, Daniela Damjanovic, Niroshan Thanthrige-Don, Mangalakumari Jeyanathan, Zhou Xing

**Affiliations:** McMaster Immunology Research Centre, and Department of Pathology & Molecular Medicine, McMaster University, Hamilton, Ontario, Canada; National Jewish Health, United States of America

## Abstract

Tuberculosis (TB) vaccine-induced airway luminal T cells (ALT) have recently been shown to be critical to host defense against pulmonary TB. However, the mechanisms that maintain memory ALT remain poorly understood. In particular, whether respiratory mucosal exposure to environmental agents such as endotoxin may regulate the size of vaccine-induced ALT population is still unclear. Using a murine model of respiratory genetic TB vaccination and respiratory LPS exposure, we have addressed this issue in the current study. We have found that single or repeated LPS exposure increases the number of antigen-specific ALT which are capable of robust secondary responses to pulmonary mycobacterial challenge. To investigate the potential mechanisms by which LPS exposure modulates the ALT population, we have examined the role of ALT proliferation and peripheral T cell recruitment. We have found that LPS exposure-increased ALT is not dependent on increased ALT proliferation as respiratory LPS exposure does not significantly increase the rate of proliferation of ALT. But rather, we find it to be dependent upon the recruitment of peripheral T cells into the airway lumen as blockade of peripheral T cell supplies markedly reduces the initially increased ALT. Thus, our data suggest that environmental exposure to airborne agents such as endotoxin has a profound modulatory effect on TB vaccine-elicited T cells within the respiratory tract. Our study provides a new, *M.tb* antigen-independent mechanism by which the respiratory mucosal anti-TB memory T cells may be maintained.

## Introduction

Global tuberculosis (TB) control is still facing major challenges today. In 2010, there were 8.8 million new TB cases and 1.4 million died from TB, with the majority of cases occurring in Africa and Asia [1]. Therefore, there is a desperate need for effective vaccination strategies for the control of TB. Currently, BCG is the only available human TB vaccine in the world. Most of the countries use BCG for routine vaccination. To date, 4 billion people and 90% of children have been vaccinated since its first administration in 1921 and mass immunization in late 1940 s and early 1950 s [2], [3]. However, it has done little to lessen the current severe situation of tuberculosis epidemic. Studies have shown that BCG fails to protect from pulmonary TB in adults due to the limited lung protection provided by parenteral BCG immunization, although it can protect children against severe disseminated TB [4]. Thus, in the past two decades, tremendous efforts have been made to develop new tuberculosis vaccination strategies including respiratory mucosal vaccination strategies [2], [3], [5].

Among the most promising TB vaccine platforms amenable to respiratory mucosal immunization are recombinant adenovirus-based TB vaccines [5], [6]. Indeed, single intranasal (i.n) administration of a recombinant human type 5 adenovirus-based vaccine expressing an *Mycobacterium tuberculosis* (*M.tb*) antigen Ag85A (AdAg85A), either alone or in combination with BCG priming, induced robust long-lasting immune protective Ag85A-specific CD8 T cell responses within the airway lumen and lung interstitium [7]–[9]. Activation of the airway luminal T cells (ALT) by i.n AdAg85A immunization was independent of CD4 T cell help [10]. The maintenance of memory ALT was found to depend on antigen-stimulated in situ proliferation but not on the continuing recruitment of peripheral T cells [8]. Of importance, different from the peripherally located vaccine-induced T cells including those located within the lung interstitium, compelling evidence indicates that the ALT are central to lung protection particularly at early stages of pulmonary *M.tb* infection [8], [9]. Since the respiratory tract is continuously exposed to the environmentally borne agents, presumably the vaccine-induced ALT are subject to the potential modulating effects by some of these agents. Mounting evidence suggests that following respiratory flu or Sendai viral infection, the memory T cells in the airway may respond to non-specific stimulation by other unrelated viral agents [11], [12]. However, thus far the vaccine-induced ALT have only been studied in naïve lungs, and the potential modulating effects from environmental agents on the ALT still remain unclear.

Based on the consideration that lipopolysaccharide (LPS) is a cell wall component of gram-negative bacteria and LPS or LPS-contaminants are ubiquitous in the environment [13]–[16], in the present study, we have elected to investigate whether respiratory exposure to LPS may modulate AdAg85A-induced ALT in a murine model, and if so, what are the potential mechanisms. We have found that respiratory mucosal LPS exposure has a potent enhancing effect on genetic TB vaccine-elicited memory T cells in the airway. Our findings provide new insights into the mechanisms by which the respiratory mucosal anti-TB memory T cells may be maintained, independent of *M.tb*-specific antigens.

## Materials and Methods

### Ethic statement

All animal experiments including animal care and procedures were conducted in accordance with the guidelines from the Canadian Council on Animal Care. This study was approved by the Animal Research Ethics Board of McMaster University with an animal utilization protocol number 10-04-23.

**Figure 1:**
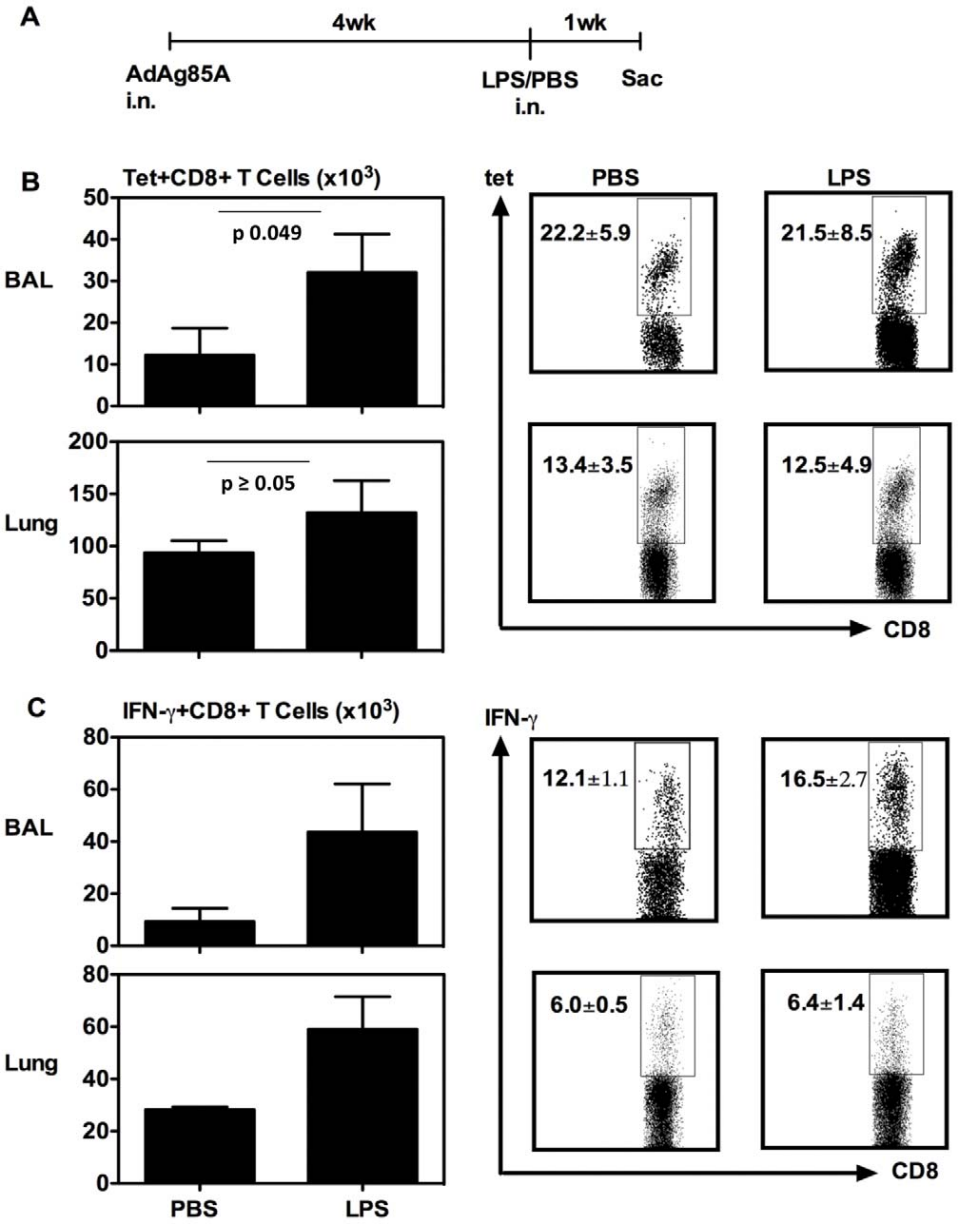
Respiratory LPS exposure increases vaccine-induced T cells in airway lumen (BAL) and lung interstitium at 7 days. (A) Experimental schema; At 7 days post-LPS or -PBS, mice were sacrificed and BAL and lung cells were isolated, and Ag85A tetramer (Tet)-specific (B) and IFN-γ-secreting (C) CD8^+^ T cells in BAL or lung interstitium were determined by immunostaining, intracellular cytokine staining and FACS. Representative dotplots are shown with the average frequency ± SEM from three animals/group. The data in graphs are expressed as mean value ± SEM of three animals per group.

**Figure 2:**
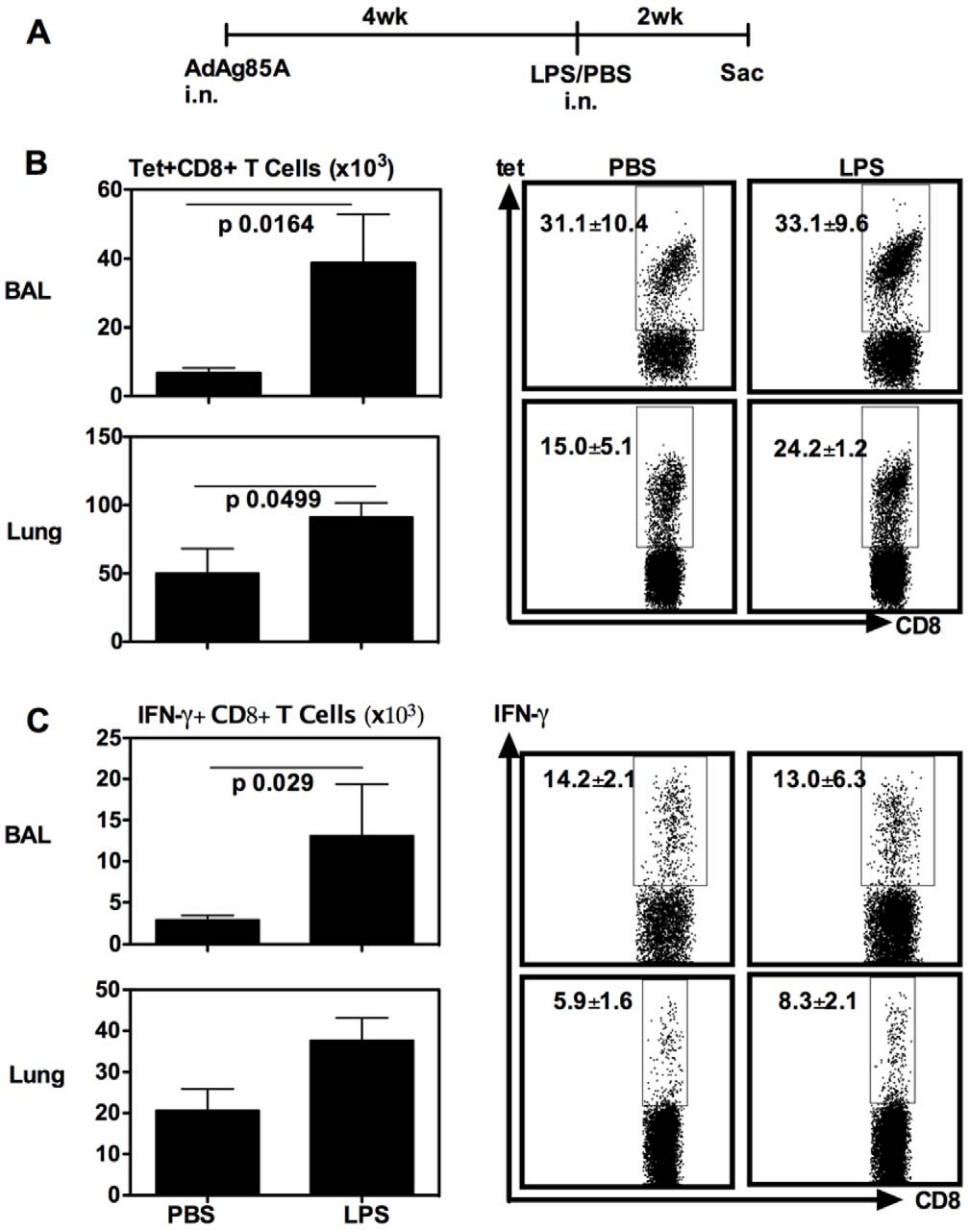
Respiratory LPS exposure increases vaccine-induced T cells in airway lumen (BAL) and lung interstitium at 14 days. (A) Experimental schema; At 14 days post-LPS or -PBS, mice were sacrificed and BAL and lung cells were isolated, and Ag85A tetramer (Tet)-specific (B) and IFN-γ-secreting (C) CD8^+^ T cells in BAL or lung interstitium were determined by immunostaining, intracellular cytokine staining and FACS. Representative dotplots are shown with the average frequency ± SEM from three animals/group. The data in graphs are expressed as mean value ± SEM of three animals per group, representative of three independent experiments.

### Mice

Female BALB/c mice (6–8 wk old) were purchased from Charles River Laboratories (Charles River, St Constant, Quebec, Canada) and housed in a specific pathogen-free, level B facility. All animals were maintained on a constant light: dark 12∶12 cycle and given free access to food and water. For all experiments, mice were euthanized by exsanguination of the abdominal artery under anesthesia.

### Intranasal viral-based TB immunization and LPS delivery

Mice were immunized intranasally (i.n) once with 5×10^7^ pfu of a recombinant replication-deficient Ad–based TB vaccine (AdAg85A) previously constructed by us [7]. Mice were administrated i.n with 25 µg of LPS (Sigma) at 4 weeks post-immunization. For repeated delivery of LPS, 14 days after the first delivery, another dose of 25 µg of LPS was delivered i.n into mice. Control mice received only PBS. At different time points after LPS delivery, mice were sacrificed and immune cells harvested.

**Figure 3:**
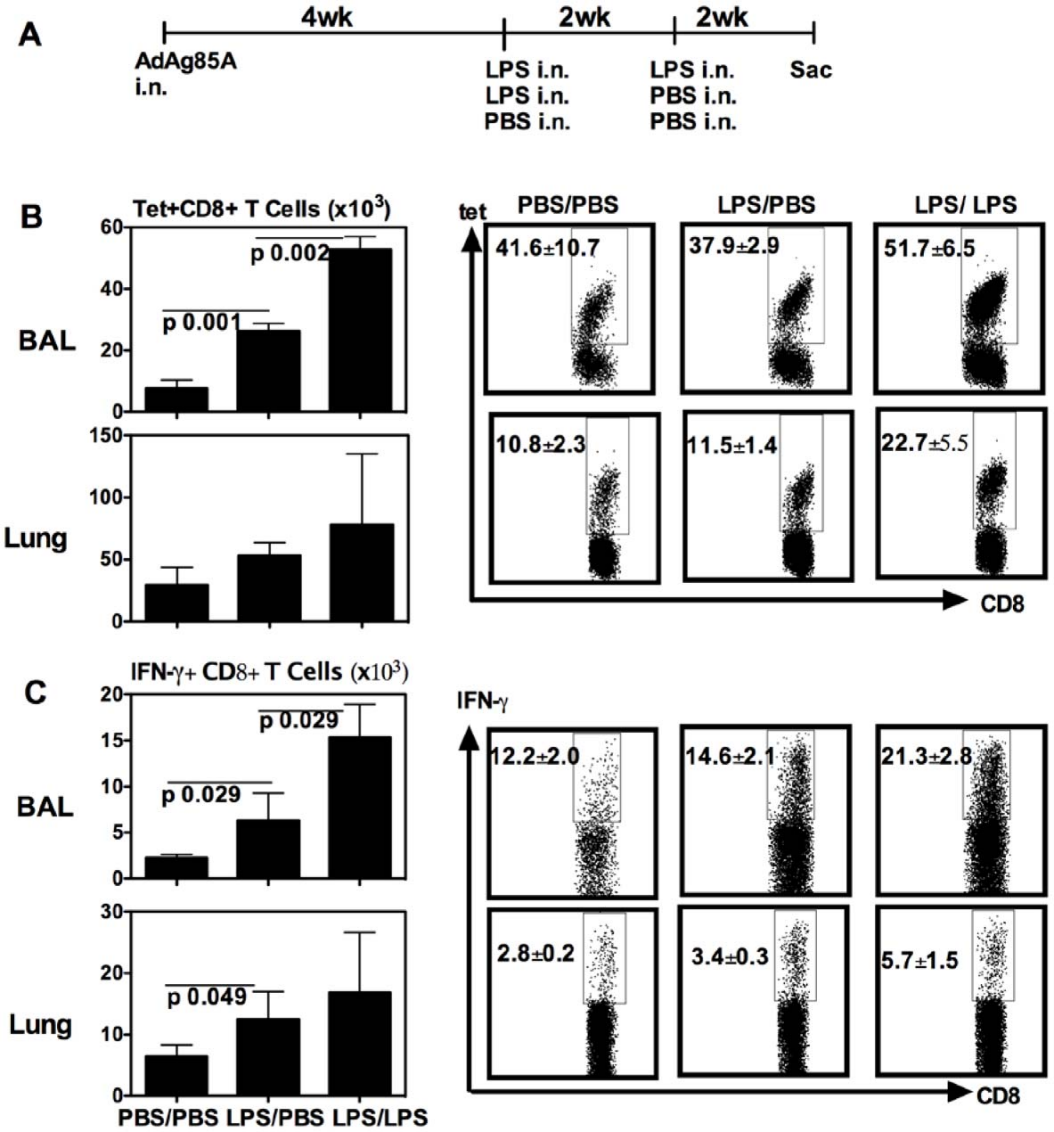
Repeated respiratory LPS exposure further increases vaccine-induced T cells in airway lumen (BAL) and lung interstitium at 14 days post-last LPS delivery. (A) Experimental schema; At 14 days post-last LPS or –PBS delivery, mice were sacrificed and BAL and lung cells were isolated, and Ag85A tetramer (Tet)-specific (B) and IFN-γ-secreting (C) CD8^+^ T cells in BAL or lung interstitium were determined by immunostaining, intracellular cytokine staining and FACS. Representative dotplots are shown with the average frequency ± SEM from three animals/group. Data in graphs are expressed as mean value ± SEM of three animals per group.

### Pulmonary mycobacterial challenge

In some experiments, vaccinated and LPS-treated mice were challenged intratracheally with 500,000 cfu of *M. tuberculosis* (H37Ra) as previously described [17]. The mice were sacrificed 7 days post-challenge for T cell analysis.

### Bronchoalveolar lavage and lung immune cell isolation

At different time points after LPS delivery, mice were sacrificed and subject to bronchoalveolar lavage (BAL), and lung tissue mononuclear cells were isolated from lavaged lung tissue as previously described [7]–[9]. Briefly, the lungs were cut into small pieces and incubated in 10 mL of 150 U/mL collagenase type I (Sigma-Aldrich, St. Louis, MO) per lung for 1 hour at 37°C, with agitation. The lung pieces were then crushed through 40 µm basket filters, red blood cells were lysed with ACK lysis buffer [0.15 mol/L NH_4_Cl, 1 mol/L KHCO_3_, 0.1 mmol/L Na_2_EDTA (pH 7.4)], and the remaining lung cells were resuspended in complete RPMI.

**Figure 4:**
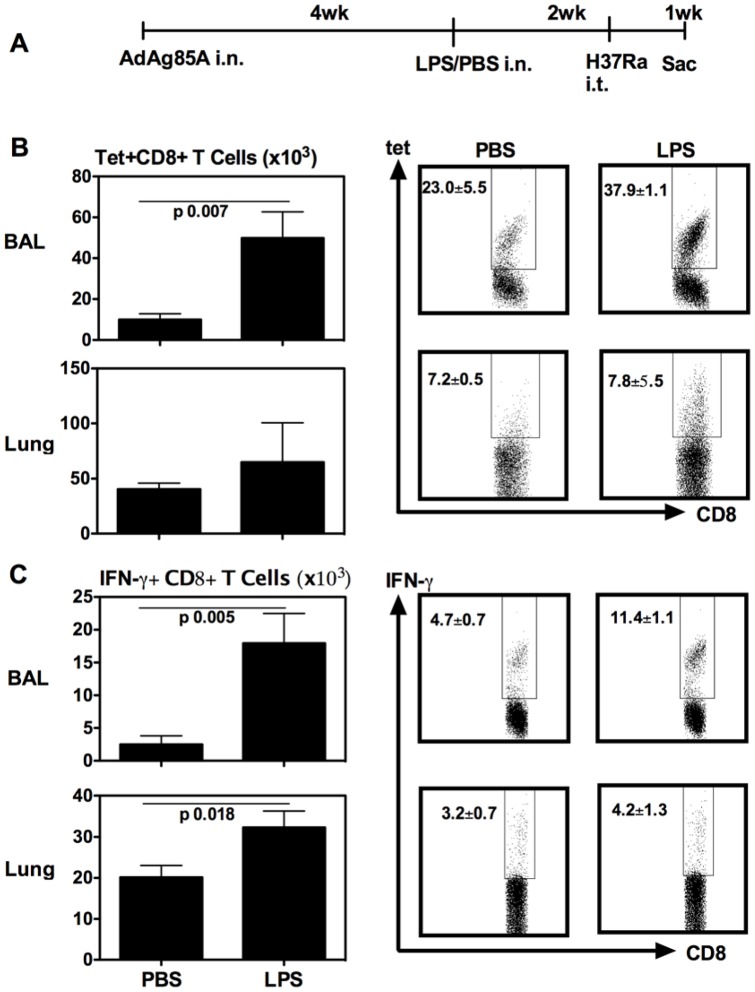
Respiratory LPS exposure-increased airway luminal T cells are able to undergo enhanced secondary T cell responses to pulmonary mycobacterial challenge. (A) Experimental schema. At 7 days post-mycobacterial challenge, mice were sacrificed and BAL and lung cells were isolated, and Ag85A tetramer (Tet)-specific (B) and IFN-γ-secreting (C) CD8^+^ T cells in BAL or lung interstitium were determined by immunostaining, intracellular cytokine staining and FACS. Representative dotplots are shown with the average frequency ± SEM from three animals/group. Data in graphs are expressed as mean value ± SEM of three animals per group.

### Cell culture, ICCS, tetramer staining, and flow cytometry

Single-cell suspensions isolated from lung were cultured in a U-bottom 96-well plate at a concentration of 20 million cells/ml, and BAL cells were plated at a concentration of 0.5 million cells/ml. Cells were cultured and stimulated for ICCS, tetramer staining, and FACS as previously described [7]–[9]. Briefly, cells were cultured in the presence of Golgi plug (5 µg/ml brefeldin A; BD Pharmingen) and with or without stimulation by Ag85A-specific CD8 (MPVGGQSST) T cell peptides at a concentration of 1 µg/well for 5–6 h [8], [9]. Cells were then washed and blocked with anti-CD16/CD32 in 0.5% BSA-PBS for 15 min on ice and immunostained with CD4a APC-Cy7, CD8a-PE-Cy7, and CD3-Pacific Blue (BD Pharmingen). Cells were then washed, permeabilized, and stained with IFN-γ-APC according to the manufacturer's instructions (BD Pharmingen). For tetramer immunostaining and FACS, a tetramer for immunodominant Ag85A CD8 T cell peptide (MPVGGQSST) bound to the BALB/c MHC class I allele H-2L^d^ ordered from the MHC Tetramer Laboratory of Baylor College of Medicine was used. Cells were washed and blocked with anti-CD16/CD32 in 0.5% BSA-PBS for 15 min on ice and stained with tetramer for 1 h in the dark at room temperature. Cells are washed and stained with surface antibodies. Stained cells were run on a LSR II (BD Pharmingen), and 100,000 events were collected per sample and analyzed with FlowJo Software.

**Figure 5:**
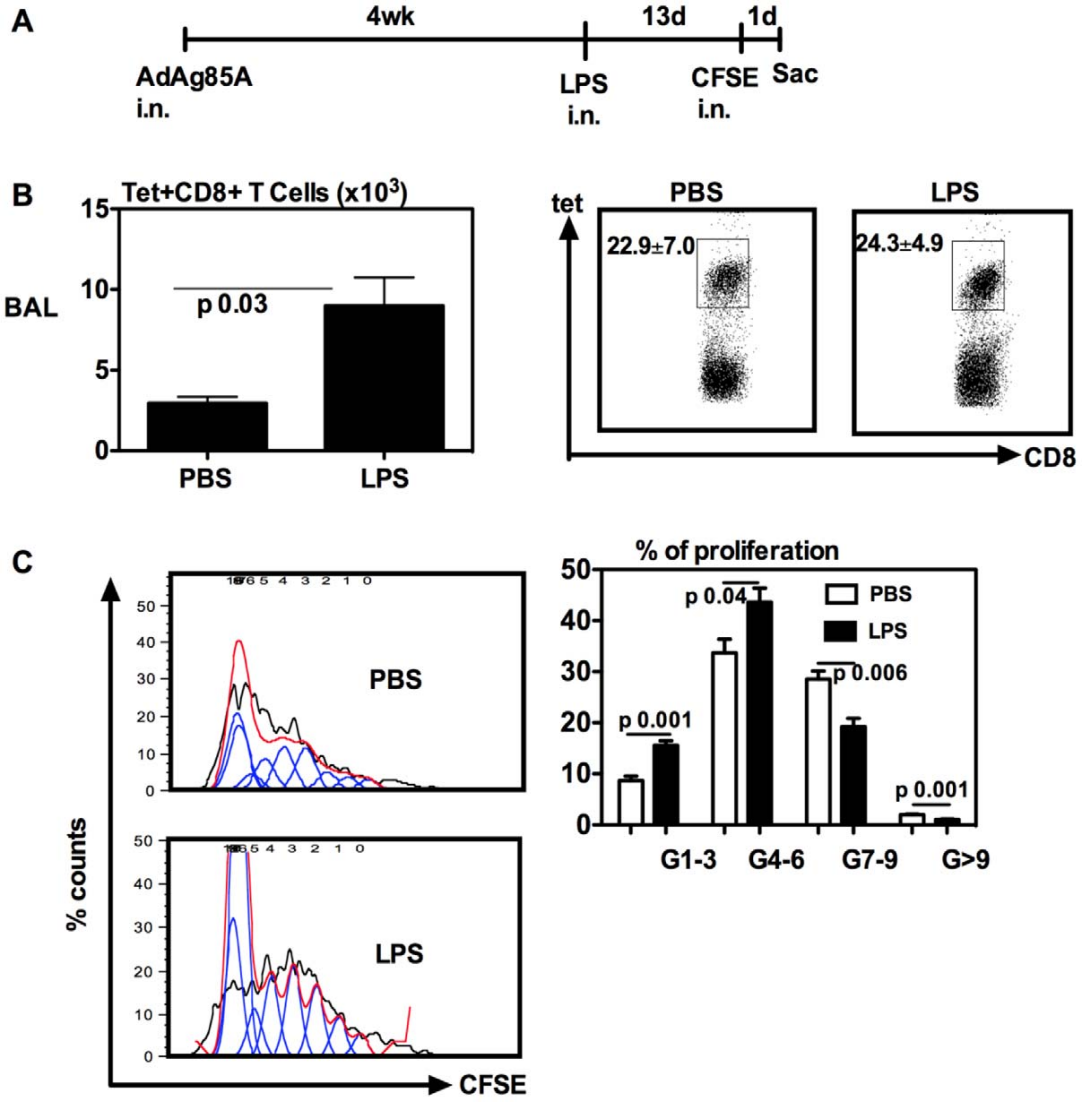
Respiratory LPS exposure does not increase the rate of proliferation of vaccine-induced airway luminal T cells. (A) Experimental schema; At 14 days post-LPS or -PBS delivery or 1 day post-CFSE delivery, mice were sacrificed and BAL cells were isolated, and Ag85A tetramer (Tet)-specific CD8 T cells (B) were determined by immunostaining and FACS. Representative dotplots are shown with the average frequency ± SEM from three animals/group. The Ag85A tetramer-specific CD8^+^ T cells were further analyzed for the rate of proliferation based on the extent of dilution of incorporated fluorochrome CFSE. The representative histograms of CFSE dilution profiles of BAL Tet^+^CD8^+^ T cells generated by using FlowJo software are shown (C left). The average percentages ± SEM from three animals/group of BAL tet^+^CD8^+^ T cells in various generations (G) of cell division are also shown (C right). Data are representative of two independent experiments.

### Cytokine and chemokine quantification

Levels of cytokines and chemokines in BAL fluids were quantified using Luminex multianalyte technology (Luminex Molecular Diagnostics, Toronto, ON, Canada), according to the manufacturer's protocols. TNF-α was determined by Elisa kit (R&D Systems).

### *In vivo* cell labeling and T cell proliferation assay

Proliferating rate of airway luminal Ag–specific CD8^+^ T cell between different groups after LPS delivery was determined by in vivo cell labeling techniques as described previously by us [8]. Briefly, mice were administered i.n with 40 µl of freshly prepared 8mM carboxyfluorescein succinimidyl ester (CFSE) and 24 h later BAL cells were harvested. As the negative control, BAL cells were harvested from PBS-treated mice. As the positive control, mice were delivered with CFSE as above but BAL cells were harvested 30 minutes after CFSE delivery. BAL cells were stained by tetramer as described above and stained with surface markers for CD3, CD8 and CD4. Stained cells were then run on a LSR II (BD Pharmingen), and 100,000 events were collected per sample. Rate of CFSE dilution in CD8+Tet^+^ T cells was determined as an index for the extent of T cell proliferation.

**Figure 6:**
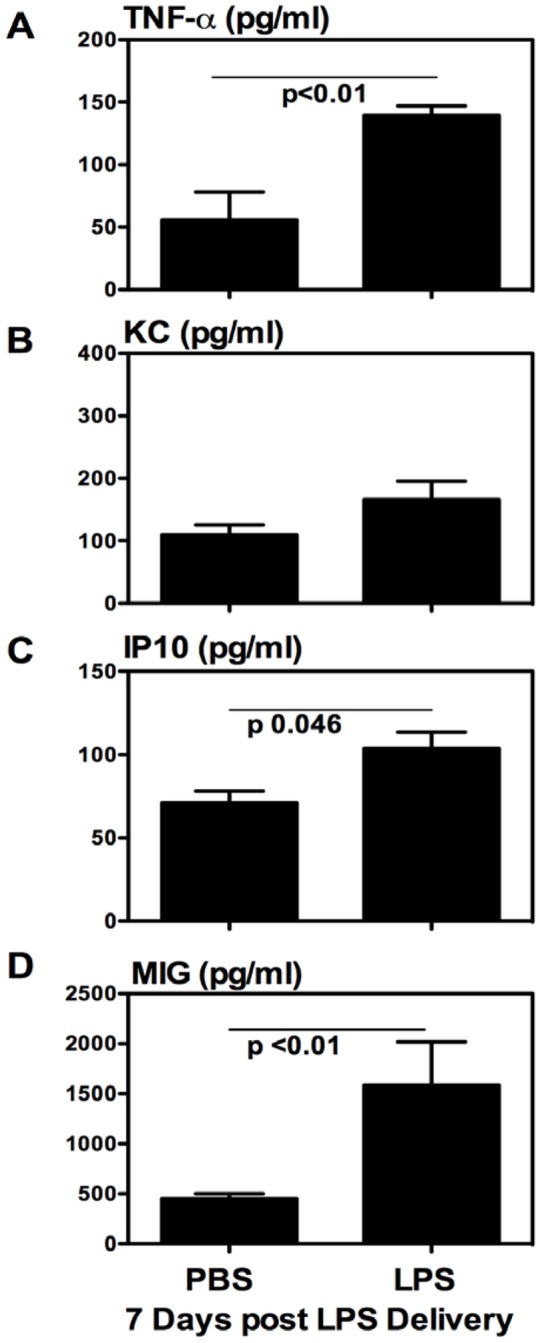
Increased levels of proinflammatory cytokines/chemokines in the airway lumen of LPS-exposed and vaccinated animals. BAL fluids were collected from the lung of the animals set up according to Fig. 1A experimental schema. Cytokine and chemokine contents were determined by Luminex or ELISA assays. Data were expressed as mean value ± SEM of three animals per group.

### Administration of FTY720

To block the peripheral T cell supply and recruitment to the airway lumen, FTY720 (Cayman Chemical, Ann Arbor, Michigan) was injected intraperitoneally in 200 µl (30% of ethanol) at 4 mg/kg body weight by repeated doses to vaccinated mice to induce and maintain the deficiency of circulating T cells as described previously by us [8]. The first dose of FTY720 was delivered one day before LPS delivery. The second and third dose of FTY720 was delivered 3 days after LPS delivery and 4 days after the second dose, respectively. The deficiency of circulating T cells was verified one day after each dose of FTY720 and on the last day of the experiment.

### Data analysis

Statistical analysis was conducted to evaluate the significance between different groups. For two-sample comparison, Student's *t* test was used. For comparison of more than two groups, ANOVA was used; wherever applicable, a *post hoc* Fisher's least significant difference test was used for further comparison. A *p* value of <0.05 was regarded as statistically significant.

**Figure 7:**
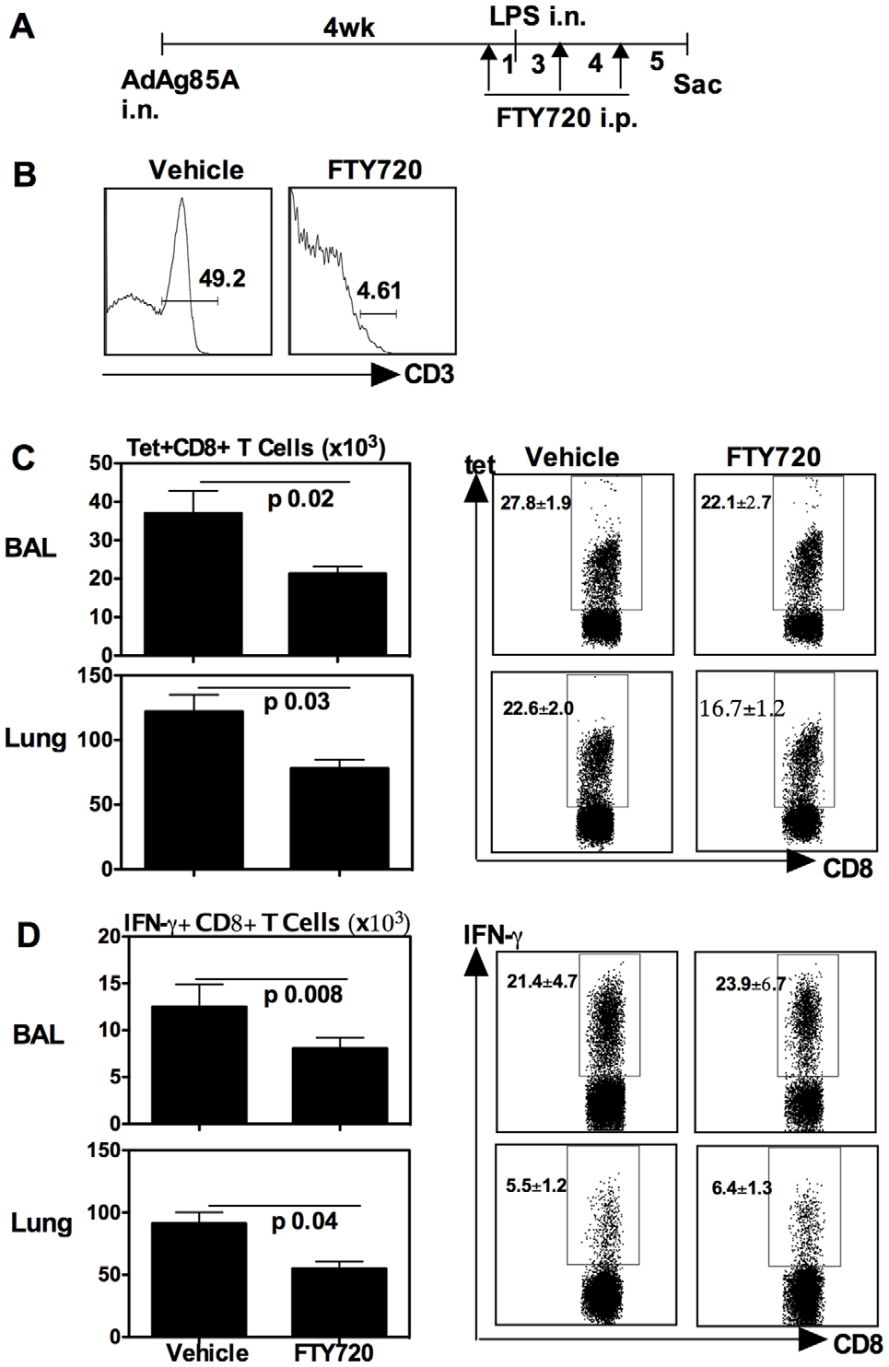
Respiratory LPS exposure-increased airway luminal T cells in the lung of vaccinated animals is dependent on the recruitment of peripheral T cells. (A) Experimental schema; At 1 day after each FTY720 delivery, the deficiency of peripheral blood T cells was verified. The representative histogram is shown (B). At 5 days post-last FTY720 or -vehicle delivery, mice were sacrificed and BAL and lung cells were isolated, and Ag85A tetramer (Tet)-specific (C) and IFN-γ-secreting (D) CD8^+^ T cells in BAL or lung interstitium were determined by immunostaining, intracellular cytokine staining and FACS. Representative dotplots are shown with the average frequency ± SEM from three animals/group. The data in graphs are expressed as mean value ± SEM of three animals per group, representative of two independent experiments.

## Results

### Respiratory LPS exposure increases vaccine-induced antigen-specific airway luminal T cells

We first investigated whether respiratory exposure to LPS had any effect on the airway luminal T cells (ALT) following respiratory immunization with AdAg85A vaccine. Thus, BALB/c mice were immunized intranasally (i.n) once with AdAg85A and 4 weeks later, a single dose of LPS was delivered i.n into the lung of these mice and the ALT and lung T cell responses in bronchoalveolar lavage (BAL) and lung interstitium (Lung) were examined first at 1 week post-LPS delivery (Fig. 1A). Both Ag85A tetramer immunostaining (Tet) and intracellular cytokine staining (ICCS) were used to evaluate the Ag85A-specific T cell responses in these lung tissue compartments. We found that compared to PBS control, respiratory LPS exposure did not markedly increase the frequencies of Tet+ or IFN-γ+ CD8 T cells either in BAL or lung interstitium (Fig. 1B/C). However, there were greater numbers of these cell populations resulting from increased total mononuclear cells in these tissue compartments (Fig. 1B/C). To examine whether the effect of single LPS exposure on the ALT and lung T cells might last, the mice were treated as in Fig. 1A except that they were sacrificed at 2 weeks post-LPS delivery (Fig. 2A). Compared to PBS, LPS exposure increased the frequencies of Tet+ and IFN-γ+ CD8 T cells in the lung interstitium but not in BAL (Fig. 2B/C). However, there were significantly much greater numbers of Tet+ and IFN-γ+ T cells found both in the BAL and lung interstitium of LPS-treated animals (Fig. 2B/C). The above data together suggest that single respiratory exposure to TLR ligands such as LPS could modulate the size of respiratory mucosal memory CD8 T cell populations following respiratory mucosal genetic TB vaccination.

### Effect of repeated respiratory LPS exposure on vaccine-induced antigen-specific airway luminal T cells

We next investigated whether over the effect by single LPS exposure, repeated respiratory LPS exposure might further increase the size of respiratory mucosal memory CD8 T cell populations following respiratory mucosal genetic TB vaccination. To this end, mice were delivered i.n with the first LPS dose at 4 weeks post-i.n AdAg85A immunization and were then given the second LPS delivery at 2 weeks post-1^st^ LPS. These mice were sacrificed at 2 weeks post-2^nd^ LPS delivery for T cell analysis (Fig. 3A). As control, some mice received only one dose of LPS or PBS (Fig. 3A). Similar to the data shown in Figure 2B/C, single LPS exposure increased the number of Tet+ and IFN-γ+ CD8 T cells both in the BAL and lung intersititium (Fig. 3B/C). In comparison, two repeated LPS exposure markedly further increased not only the frequencies but also the numbers of these T cell population primarily in the BAL compartment (Fig. 3B/C). The frequencies of Tet+ and IFN-γ+ CD8^+^ T cells in the BAL were significantly different between one dose LPS- and two-dose LPS-treated groups of mice (p = 0.029 LPS/PBS vs LPS/LPS BAL Tet+; p = 0.028 LPS/PBS vs. LPS/LPS BAL IFN-γ+ T cells). There was also an increased trend in the number of lung interstitial T cells by repeated LPS exposure but the difference in absolute numbers was not significant (Fig. 3B/C). These data suggest that repeated LPS exposure has a profound enhancing effect on TB vaccine-induced mucosal T cell populations, particularly the ALT.

### Effect of respiratory LPS exposure on secondary responses of vaccine-induced airway luminal T cells to pulmonary mycobacterial challenge

To examine the ability of vaccine-induced ALT after LPS exposure to respond to pulmonary mycobacterial challenge, at 2 weeks after single LPS delivery to AdAg85A-immunized mice, mice were challenged via the airway with mycobacteria (*M.tb*H37Ra). The mice were then sacrificed and T cell responses examined at 7 days post-mycobacterial challenge (Fig. 4A). Compared to mock-challenged control animals, LPS-exposed animals mounted a much greater T response in BAL to pulmonary mycobacterial challenge. The frequencies of both Tet+ and IFN-γ+ CD8 T cells in BAL of LPS-exposed animals were significantly greater than in PBS controls (Fig. 4B/C). Furthermore, the absolute numbers of these cells were significantly greater in BAL of LPS-exposed animals than in PBS controls (Fig. 4B/C). In general, LPS-exposed animals also mounted higher levels of T cell responses in the lung interstitium but they were less dramatic than those in BAL. Together, these data suggest that the vaccine-induced airway luminal T cells modulated by respiratory LPS exposure are able to vigorously respond to pulmonary mycobacterial infection.

### Effect of respiratory LPS exposure on the proliferation of vaccine-induced airway luminal T cells

To begin investigating the mechanisms by which respiratory LPS exposure increased the ALT, we first examined the rate of proliferation of the ALT with or without respiratory LPS exposure. To this end, we employed a lung CFSE labeling approach that we have previously established [8]. This approach allows us to fluorescently label all airway luminal cells in vivo and examine the extent of airway luminal T cell proliferation ex vivo by assessing the rate of CFSE dilution within the labeled antigen-specific T cells. Thus, mice were immunized i.n with AdAg85A and at 4 weeks post-immunization, they were delivered i.n with LPS. At day 13 post-LPS, these mice were given i.n with CFSE. Mice were then sacrificed and the ALT analyzed at 24 h after CFSE delivery (Fig. 5A). Once again, respiratory LPS exposure significantly increased the number of Tet+ CD8 T cells in the airway lumen (Fig. 5A). Upon comparing the rate of airway luminal Tet+ CD8 T cell proliferation, we found that compared to the control treatment, respiratory LPS exposure led to increased rates of the proliferation of T cells that had undergone 1 to 3, and 4 to 6 generations/rounds of proliferation (Fig. 5C). For instance, the average percentages of proliferating ATL in G1–3 between control and LPS exposure groups are 8.5% and 15.4%, respectively. However, there were greater frequencies of proliferating ATL in the control group that had undergone 7 to 9 or more rounds of proliferation (Fig. 5C). Thus, overall about 65% of the ALT in control animals underwent 4 to 9 rounds of proliferation while 59% of the ALT in LPS-exposed animals underwent only 1 to 6 rounds of proliferation. These data together suggest that consistent with our previous findings [8], the vaccine-induced ALT are highly proliferative but on the other hand, the moderately altered proliferation rate of the ALT by respiratory LPS exposure is unlikely the reason for the increased size of the ALT population by LPS.

### Respiratory LPS exposure increases proinflammatory cytokine and chemokine responses in the airway lumen

To further examine the mechanisms by which respiratory LPS exposure increased the ALT, we determined the levels of TNF-α and chemokines KC, IP-10 and MIG in BAL collected at 7 days after LPS delivery. The levels of TNF-α in the BAL of LPS-exposed animals were found to be significantly elevated over those of control animals (Fig. 6A). In comparison, the levels of neutrophil chemokine KC were comparable between control and LPS-exposed animals (Fig. 6B). However, we found that the levels of mononuclear cell-recruiting chemokines IP-10 and MIG in the BAL of LPS-exposed animals were markedly higher than those in the control animals (Fig. 6C/D). These data supports the possibility that LPS exposure increases the size of vaccine-induced ALT population via enhancing the recruitment of peripheral T cells.

### Respiratory LPS exposure increases vaccine-induced antigen-specific airway luminal T cells via enhancing peripheral T cell recruitment

To examine whether respiratory LPS exposure indeed increased vaccine-induced antigen-specific airway luminal T cells via enhancing peripheral T cell recruitment, we employed an approach that we used previously [8] by which FTY720, a sphingosine 1-phosphate receptor modulator, was injected to induce peripheral lymphopenia and block lymphocyte trafficking. FTY720 was injected i.p one day before LPS exposure and was repeated at 3 and 7 days after LPS exposure, and the mice were sacrificed for T cell analysis 5 days post-last FTY720 injection (Fig. 7A). As control, some mice only received the vehicle injection. FTY720 efficacy was verified 1 day after each dose of delivery and right before sacrifice, and was found to consistently cause >90% decline in the circulating T cells (Fig. 7B). As shown in Fig. 7C, FTY720 treatment significantly decreased the number of antigen-specific Tet+ (Fig. 7C) and IFN-γ+ (Fig. 7D) CD8^+^ T cells in the airway lumen (BAL) of LPS-exposed and FTY720-treated animals, compared to LPS-exposed group without FTY720 treatment (Vehicle). The number of such T cells was also decreased in the lung interstitium of LPS-exposed and FTY720-treated animals (Fig. 7C/D). These data indicate that the continuing recruitment to the airway lumen of peripheral T cells represents a main mechanism for increased antigen-specific airway luminal CD8^+^ T cells by respiratory LPS exposure.

## Discussion

Our study demonstrates that single or repeated respiratory mucosal exposure to endotoxin has a potent regulatory effect on genetic TB vaccine-induced memory T cells in respiratory mucosal tissue. This modulatory effect is particularly prominent on vaccine-induced airway luminal T cells (ALT) that reside on the surface of respiratory mucosa. LPS exposure significantly increases the number of ALT what are not only Ag85A tetramer-specific but also IFN-γ-producing upon ex vivo antigen re-stimulation. Such effect by a single LPS exposure begins from 3 days and can still be readily appreciated 2 weeks post-exposure, and is able to translate to enhanced secondary effector ALT responses upon pulmonary mycobacterial challenge. In addition to LPS, we have also seen a similar effect by respiratory exposure to a different TLR agonist, PGN (data not shown). Such increases by LPS exposure in the size of vaccine-induced ALT population cannot be accounted for by altered in situ T cell proliferation as we find the ALT in LPS-exposed animals not to undergo an accelerated rate of proliferation compared to the control. Rather, we find that significantly increased ALT by LPS exposure is due to enhanced recruitment into the airway of peripheral T cells since the blockade of peripheral T cell supplies, by using a FTY720 approach, was found to significantly diminish the initially LPS-increased ALT population.

To date, whether the vaccine-induced ALT may be subject to the modulation by respiratory exposure to environmental agents, has remained unclear. Overall, the data from our study suggest that environmental exposure to airborne agents such as endotoxin may have a profound modulatory effect on TB vaccine-elicited memory T cells within the respiratory tract. Our study thus provides a novel, *M.tb* antigen-independent mechanism by which the respiratory mucosal anti-TB memory T cells may be maintained. We have recently shown that the long-term maintenance of vaccine-induced ALT is dependent on continuing in situ proliferation and specific *M.tb* antigen expressed by the vaccine, and can be independent of the recruitment of peripheral T cells [8]. These previous findings, with our current data, indicate that the *M.tb* antigen-driven and the *M.tb* antigen-independent regulation of the ALT may operate via different mechanisms. Furthermore, these findings together suggest that in real life situations as opposed to the SPF environment where experimental mice are kept, the memory ALT on the surface of respiratory mucosa are regulated and maintained by both *M.tb* antigens and nonspecific environmental agents. Given the recently well-recognized importance of lung T cells, particularly ALT, rather than the peripheral T cells, in pulmonary protection against TB [3], [18], [19], the findings presented in our current study hold important implications. It has been increasingly recognized that respiratory mucosal vaccination may elicit the most robust immune protection against pulmonary TB [2], [3], [5]. Among a dozen of promising candidate TB vaccines currently in human studies is the genetic TB vaccine AdAg85A tested in the current study that has been undergoing early phases of clinical investigation following intramuscular vaccination in human subjects in Canada [20]. Enhanced knowledge in how the ALT triggered by mucosal AdAg85A vaccination are regulated will provide much needed information to enable us to properly design and carry out human studies by respiratory mucosal AdAg85A vaccination in near future. Respiratory mucosal AdAg85A vaccination may help bypass the undesired effects imposed by pre-existing anti-adenovirus antibodies as most of human populations have such pre-existing anti-Ad5 immunity [21], [22].

In our current study we have observed what is considered a lasting pro-inflammatory effect in the airway lumen following a single respiratory LPS exposure as significantly raised levels of cytokines including TNF-α, IP-10 and MIG were found in the BAL even 7 days post-LPS delivery. This observation contrasts the transient acute inflammatory responses that are seen following lung LPS delivery to naïve rodent lung [23]. The persistently raised levels of cytokine responses observed in our current study is likely due to differential responses to LPS exposure in a vaccine-primed lung environment. Such proinflammatory environment possesses the chemotactic signals continuously drawing the peripheral Ag85A-specific T cells into the airway lumen and lung intersititum even at 2 weeks after LPS exposure. To support our observations, previous studies have shown that chemokines such as MIG (CXCL9) and IP-10 (CXCL10) play an essential role in directing anti-Sendai or respiratory syncytial virus memory T cells into the respiratory tract [12], [24]. Indeed, our findings that FTY720-mediated blockade of peripheral T cell supplies obliterated LPS exposure-increased ALT, support this functional linkage. On the other hand, we find the potential stimulating effect by LPS on T cell proliferation to be minimal. This finding does not support the view that intravenous LPS injection may stimulate T cell proliferation via an indirect pathway involving APC stimulation and release of type I interferons [25].

In conclusion, we have observed that respiratory mucosal exposure to TLR agonists such as endotoxin has a potent enhancing effect on respiratory mucosal memory T cells induced by respiratory mucosal genetic-based TB vaccination. Such effect is mediated primarily via increased recruitment into the airway lumen of peripheral T cells. Our findings provide a novel, *M.tb* antigen-independent mechanism by which respiratory mucosal memory T cells are maintained. It is our belief that the implication of these findings goes beyond the immunobiology of mucosal TB vaccination as such T cells are also present in the airway lumen of humans previously exposed to *M.tb* or other mycobacterial species [26].

## References

[1] Lawn SD, Zumla AI (2011). Tuberculosis.. Lancet.

[2] Kaufmann SH, Hussey G, Lambert PH (2010). New vaccines for tuberculosis.. Lancet.

[3] Jeyanathan M, Heriazon A, Xing Z (2010). Airway luminal T cells: a newcomer on the stage of TB vaccination strategies.. Trends Immunol.

[4] Trunz BB, Fine P, Dye C (2006). Effect of BCG vaccination on childhood tuberculous meningitis and miliary tuberculosis worldwide: a meta-analysis and assessment of cost-effectiveness.. Lancet.

[5] Xing Z, Lichty BD (2006). Use of recombinant virus-vectored tuberculosis vaccines for respiratory mucosal immunization.. Tuberculosis.

[6] Lasaro MO, Ertl HC (2009). New insights on adenovirus as vaccine vectors.. Mol Ther.

[7] Wang J, Thorson L, Stokes RW, Santosuosso M, Huygen K (2004). Single mucosal, but not parenteral, immunization with recombinant adenoviral-based vaccine provides potent protection from pulmonary tuberculosis.. J Immunol.

[8] Jeyanathan M, Mu J, McCormick S, Damjanovic D, Small CL (2010). Murine airway luminal antituberculosis memory CD8 T cells by mucosal immunization are maintained via antigen-driven in situ proliferation, independent of peripheral T cell recruitment.. Am J Respir Crit Care Med.

[9] Santosuosso M, Zhang X, McCormick S, Wang J, Hitt M (2005). Mechanisms of mucosal and parenteral tuberculosis vaccinations: adenoviral-based mucosal immunization preferentially elicits sustained accumulation of immune protective CD4 and CD8 T cells within the airway lumen.. J Immunol.

[10] Mu J, Jeyanathan M, Shaler CR, Horvath C, Damjanovic D (2010). Respiratory mucosal immunization with adenovirus gene transfer vector induces helper CD4 T cell-independent protective immunity.. J Gene Med.

[11] Ely KH, Cauley LS, Roberts AD, Brennan JW, Cookenham T (2003). Nonspecific recruitment of memory CD8+ T cells to the lung airways during respiratory virus infections.. J Immunol.

[12] Kohlmeier JE, Woodland D (2006). Memory T cell recruitment to the lung airways.. Curr Opin Immunol.

[13] Beutler B, Rietschel ET (2003). Innate immune sensing and its roots: the story of endotoxin.. Nat Rev Immunol.

[14] Nilsson A, Kihlström E, Lagesson V, Wessén B, Szponar B (2004). Microorganisms and volatile organic compounds in airborne dust from damp residences.. Indoor Air.

[15] Park JH, Szponar B, Larsson L, Gold DR, Milton DK (2004). Characterization of lipopolysaccharides present in settled house dust.. Appl Environ Microbiol.

[16] Larsson L, Szponar B, Pehrson C (2004). Tobacco smoking increases dramatically air concentrations of endotoxin.. Indoor Air.

[17] Horvath CN, Shaler CR, Jeyanathan M, Zganiacz A, Xing Z (2012). Mechanisms of delayed anti-tuberculosis protection in the lung of parenteral-BCG vaccinated hosts: A critical role of airway luminal T cells.. Mucosal Immunol.

[18] Urdahl KB, Shafiani S, Ernst JD (2011). Initiation and regulation of T-cell responses in tuberculosis.. Mucosal Immunol.

[19] Cooper AM (2009). T cells in mycobacterial infection and disease.. Curr Opin Immunol.

[20] Brennan MJ, Thole J (2012). Tuberculosis vaccines: a strategic blueprint for the next decade.. Tuberculosis (Edinb).

[21] Sumida SM, Truitt DM, Kishko MG, Arthur JC, Jackson SS (2004). Neutralizing antibodies and CD8+ T lymphocytes both contribute to immunity to adenovirus serotype 5 vaccine vectors.. J Virol.

[22] Mast TC, Kierstead L, Gupta SB, Nikas AA, Kallas EG (2010). International epidemiology of human pre-existing adenovirus (Ad) type-5, type-6, type-26 and type-36 neutralizing antibodies: correlates of high Ad5 titers and implications for potential HIV vaccine trials.. Vaccine.

[23] Xing Z, Kirpalani H, Torry D, Jordana M, Gauldie J (1993). Polymorphonuclear leukocytes as a significant source of tumor necrosis factor-alpha in endotoxin-challenged lung tissue.. Am J Pathol.

[24] Ely KH, Cookenham T, Roberts AD, Woodland DL (2006). Memory T cell populations in the lung airways are maintained by continual recruitment.. J Immunol.

[25] Tough DF, Sun S, Sprent J (1997). T Cell Stimulation In Vvo by Lipopolysaccharide (LPS).. J Exp Med.

[26] Walrath JR, Silver RF (2011). The α4β1 integrin in localization of Mycobacterium tuberculosis-specific T helper type 1 cells to the human lung.. Am J Respir Cell Mol Biol.

